# The Epidemiology and Socioeconomic Associates of Pulmonary Aspiration and Foreign Body in the Airway in the Middle East and North Africa Region From 1990 to 2021: A Descriptive Epidemiological Study

**DOI:** 10.1002/hsr2.72311

**Published:** 2026-05-09

**Authors:** Fereshteh Kimia, Fatemeh Roodneshin, Mahtab Poorzamany Nejat Kermany, Hamidreza Samadpour, Nima Saeedi, Mina Vishte

**Affiliations:** ^1^ Department of Anesthesiology, Labbafinejad Hospital Shahid Beheshti University of Medical Sciences Tehran Iran; ^2^ Department of Anesthesiology Shahid Beheshti University of Medical Sciences Tehran Iran; ^3^ Labbafinejad Hospital Shahid Beheshti University of Medical Sciences Tehran Iran; ^4^ Shahid Beheshti University of Medical Sciences Tehran Iran

**Keywords:** disability‐adjusted life years, foreign body, global burden of disease, North Africa and Middle East, respiratory aspiration

## Abstract

**Background and Aims:**

Pulmonary aspiration and foreign body in the airway (PAFBA) is one of the medical emergencies, which can mostly affect children and geriatrics. Reporting the epidemiology of this condition is necessary to plan preventive and controlling measures. Our objective was to investigate the burden of PAFBA in the Middle East and North Africa (MENA) region and its 21 countries from 1990 to 2021, with age and sex patterns. Moreover, the relationship with the socio‐demographic index (SDI) and the PAFBA burden was evaluated.

**Methods:**

We extracted incidence, prevalence, disability‐adjusted life years (DALYs), and death counts and rates of PAFBA for MENA and countries from the Global Burden of Disease 2021 study. The association with SDI was investigated using the smoothing spline model. The raw frequencies and age‐standardized rates were represented with 95% uncertainty intervals (UIs).

**Results:**

In 2021, age‐standardized PAFBA rates per 100,000 in the MENA region were as follows: incidence at 15.0 (11.8, 19.1), prevalence at 23.8 (19.7, 28.6), DALYs at 38.5 (23.0, 47.5), and mortality at 0.6 (0.4, 0.8), all showing declines since 1990. Lebanon and Afghanistan reported the highest age‐standardized incidence and prevalence rates, while Afghanistan and Sudan had the highest DALY and mortality rates. The under‐five age group showed the greatest burden in incidence, DALYs, and deaths, with males more affected than females. A negative association with SDI was observed.

**Conclusions:**

Despite a decline in PAFBA burden over the past 32 years in the MENA region, mortality rates and DALYs remain high. Prioritizing public awareness programs and improving diagnostic and treatment methods are essential to further reduce this burden.

## 1 Introduction

Pulmonary aspiration and foreign body in the airway (PAFBA) is a life‐threatening condition that can be categorized by patient age, type of foreign body, and site of impaction. Cases are also classified by obstruction level, with partial or complete blockages, and by origin, as either internal (e.g., mucus plugs) or external (e.g., food particles like peanuts) [1]. PAFBA has various clinical presentations in children and adults, with children often experiencing airway obstruction and respiratory distress, while adults may have distal airway involvement. Common symptoms in children include cough, wheezing, and recurrent infections, and in adults, aspiration is often asymptomatic, which can lead to potential complications such as obstructive emphysema and bronchiectasis [2]. PAFBA can also impose an economic burden on healthcare systems, particularly in less developed countries [3]. PAFBA can lead to a range of pulmonary syndromes, including both airway and parenchymal disorders, with risk increased in individuals with impaired airway defenses, dysphagia, or reflux [4].

Globally, the age‐standardized rates of incidence and death for foreign body aspiration decreased over the period 1990–2019, while the number of incident cases increased within this period [5]. Similarly, the age‐standardized incidence rate of foreign body aspiration decreased in the Middle East and North Africa (MENA) region, while it was lower than the global rate [5]. The incidence rates of foreign body aspiration were higher in males, and most deaths occurred in children and the elderly [5]. Areas with lower socio‐economic status had higher incidence rates of foreign body aspiration [5].

PAFBA is an important health concern in the MENA region, particularly among young children. Although it is rarer in adults, PAFBA has become more frequent in women in MENA who use metallic pins to secure headscarves. These cultural practices and regional use of specific objects contribute to a unique pattern of PAFBA cases, highlighting the importance of tailored healthcare approaches and preventive measures in this region. In addition, updated epidemiological data can inform healthcare policymakers about the contributing factors, so that they can develop targeted prevention and intervention strategies that address the specific needs of the MENA population [6].

Previous studies have reported the burden of foreign body aspiration at the global level or in specific populations, such as children aged less than 5 years [5, 7]. These studies reported the burden of foreign body aspiration from 1990 to 2019, using the Global Burden of Disease (GBD) study data. However, they did not focus on the MENA region and its countries. In addition, they used 2019 data, which needs to be updated. Consequently, we aimed to investigate the burden of PAFBA in MENA and its countries from 1990 to 2021, reporting the age and sex patterns. Moreover, the relationship between the socio‐demographic index (SDI) and the burden of PAFBA was assessed.

## 2 Methods

### 2.1 Overview

For this study, data on incidence, prevalence, disability‐adjusted life years (DALYs), and deaths due to PAFBA were extracted from the Global Health Data Exchange for the GBD 2021 project at https://vizhub.healthdata.org/gbd-results/. The GBD framework offers evaluations of mortality and morbidity measured with different metrics by variables such as cause, age, sex, year, and location. In this study, we specifically focused on the MENA region and its 21 countries (i.e., Afghanistan, Algeria, Bahrain, Egypt, Iran, Iraq, Jordan, Kuwait, Lebanon, Libya, Morocco, Oman, Palestine, Qatar, Saudi Arabia, Sudan, the Syrian Arab Republic, Tunisia, Turkey, the United Arab Emirates, and Yemen), using data from 1990 to 2021 [8, 9].

### 2.2 Case Definition and Data Sources

In the GBD 2021 framework, diseases and injuries are structured into four levels within a hierarchical system. Level 1 contains the COVID‐19 pandemic impact and three main groups of communicable diseases, non‐communicable diseases, and injuries. Level 2 divides these into 22 cause clusters. Level 3 specifies 175 individual causes, including 132 distinct conditions and 43 clusters that lead into the Level 4 breakdown. Level 4 further disaggregates these into 302 specific causes, and PAFBA is in Level 4 within a Level 3 injury category specifically focused on foreign body aspiration. The International Classification of Disease (ICD) codes were determined and mapped for each cause and injury. For PAFBA, relevant cases were identified using the ICD‐10 codes W75‐W76.9, W78‐W80.9, W83‐W84.9, and ICD‐9 codes 770.1‐770.18, E911‐E912.09, E913.8‐E913.99. For PAFBA, the types of data sources included vital registrations, verbal autopsy, and minimally invasive thoracic surgery diagnosis [9].

### 2.3 Modeling Strategy

The Cause of Death Ensemble model (CODEm) was used to estimate deaths due to PAFBA. CODEm is a comprehensive tool that tests and integrates various models to enhance predictive accuracy. For non‐fatal outcomes, the Bayesian meta‐regression tool DisMod‐MR 2.1 was applied, which combined data from diverse epidemiological studies. This modeling framework adjusted for recognized biases and included covariates relevant to PAFBA, such as average years of education per capita, alcohol consumption per capita, the healthcare access and quality index, per capita income, and SDI [8].

### 2.4 Statistical Analysis

We reported counts and age‐standardized rates of incidence, prevalence, DALYs, and death. DALYs were determined by summing years of life lost (YLLs) and years lived with disability (YLDs). YLLs were estimated by multiplying the number of deaths in each age group by the remaining life expectancy for that group, based on the GBD standard life table. YLDs were derived by applying disability weights to the prevalence. To account for uncertainty, we used 500 iterations for each computational step, with uncertainty intervals (UIs) defined as the 25th and 975th values of the ordered iterations. These 95% UIs reflect propagated uncertainty from data inputs, model adjustments, and parameter variability, rather than standard errors from sampling. In regions like MENA, where data sources (e.g., vital registrations and verbal autopsies) may be sparse or inconsistent due to socioeconomic and infrastructural challenges, UIs tend to be wider, indicating greater estimation uncertainty. Overlapping UIs in figures do not imply statistical non‐significance, as the GBD framework emphasizes descriptive trends and point estimates over formal hypothesis testing; however, they highlight areas where data quality improvements could refine precision. Additionally, we used smoothing spline models to examine the relationship between SDI and the burden of PAFBA across various countries. SDI values range from 0 (least developed) to 1 (most developed) and incorporate metrics such as income per capita, average years of schooling for individuals aged 15 and older, and fertility rates for those under 25. All analyses were prespecified with no exploratory or subgroup analyses conducted. All analyses were conducted using R software (version 4.2.1).

## 3 Results

### 3.1 Epidemiology at the Middle East and North Africa Region

In 2021, the incidence of PAFBA in the MENA region was 94,812 cases (95% UI: 73,968 to 120,724), with an age‐standardized incidence rate of 15.0 per 100,000 (11.8 to 19.1), marking a significant 38.9% decrease in the rate since 1990. The prevalence of PAFBA was estimated at 145,295 cases (119,328 to 175,322) with an age‐standardized point prevalence of 23.8 per 100,000 (19.7 to 28.6), reflecting a 40.9% reduction in the point prevalence of PAFBA from 1990 to 2021. DALYs attributed to PAFBA in the region reached 231,468 (138,613 to 285,866), with an age‐standardized rate of 38.5 per 100,000 (23.0 to 47.5), showing a decline of 61.8% since 1990. Additionally, the number of deaths due to PAFBA in 2021 was 3,524 (2,079 to 4,301), with an age‐standardized mortality rate of 0.6 per 100,000 (0.4 to 0.8), representing a decrease of 55.6% from 1990 (Table 1).

**Table 1:** Prevalent cases, incident cases, deaths, and DALYs, along with their age‐standardized rates, due to pulmonary aspiration and foreign body in the airway in 2021 for both sexes, and the percentage change in rates per 100,000 population from 1990 to 2021 in Middle East and North Africa.

Location	Incidence (95% UI)			Prevalence (95% UI)			DALYs (95% UI)			Deaths (95% UI)		
	Counts (2021)	Rate (2021)	Pcs in rate 1990–2021	Counts (2021)	Rate (2021)	Pcs in rate 1990–2021	Counts (2021)	Rate (2021)	Pcs in rate 1990–2021	Counts (2021)	Rate (2021)	Pcs in rate 1990–2021
**Middle East and North Africa**	94812.3 (73967.7, 120724.1)	15.0 (11.8, 19.1)	−38.9 (−43.0, −35.3)	145295.1 (119328.3, 175321.6)	23.8 (19.7, 28.6)	−40.9 (−44.6, −37.7)	231468.0 (138612.9, 285865.5)	38.5 (23.0, 47.5)	−61.8 (−68.8, −48.4)	3523.7 (2079.1, 4301.3)	0.6 (0.4, 0.8)	−55.6 (−63.0, −42.5)
**Afghanistan**	7154.9 (5495.9, 9288.4)	15.5 (12.3, 19.6)	−37.6 (−46.2, −28.3)	6921.1 (5654.4, 8329.7)	26.7 (22.1, 31.7)	−36.5 (−41.9, −30.9)	37471.8 (20973.6, 51997.2)	83.2 (48.1, 114.1)	−55.7 (−66.7, −19.7)	469.1 (264.4, 645.8)	1.3 (0.8, 1.8)	−50.3 (−61.3, −18.9)
**Algeria**	7682.3 (5937.1, 10007.5)	16.9 (13.1, 21.9)	−31.0 (−38.0, −24.5)	11497.7 (9366.9, 13927.1)	26.5 (21.6, 32.0)	−34.2 (−37.7, −30.9)	15586.6 (9337.1, 20043.3)	35.5 (21.2, 45.5)	−63.1 (−73.6, −47.2)	234.1 (138.5, 294.9)	0.6 (0.4, 0.8)	−54.2 (−65.5, −38.7)
**Bahrain**	181.6 (138.3, 234.9)	15.6 (11.8, 20.8)	−36.0 (−42.1, −29.8)	389.3 (317.2, 473.2)	24.1 (19.7, 29.2)	−39.7 (−43.3, −35.5)	252.1 (159.5, 324.3)	20.0 (13.2, 26.0)	−59.5 (−67.6, −45.2)	4.5 (2.7, 5.7)	0.5 (0.3, 0.6)	−53.0 (−62.3, −34.5)
**Egypt**	17039.5 (13035.9, 22508.2)	14.1 (11.0, 18.4)	−37.9 (−43.9, −32.7)	22230.7 (18277.2, 26833.1)	23.2 (19.3, 27.8)	−39.7 (−43.9, −35.9)	23629.7 (17244.7, 32792.4)	21.1 (15.6, 29.2)	−78.8 (−84.3, −60.4)	340.5 (248.9, 488.8)	0.4 (0.3, 0.6)	−79.0 (−84.3, −50.9)
**Iran**	9751.1 (7501.8, 12448.1)	13.6 (10.4, 17.5)	−50.2 (−53.7, −46.9)	18737.3 (15453.9, 22449.3)	21.2 (17.4, 25.4)	−52.2 (−56.1, −48.5)	17192.8 (11053.7, 21881.0)	22.7 (14.6, 29.0)	−79.4 (−84.3, −68.1)	320.9 (197.9, 408.5)	0.4 (0.3, 0.5)	−69.8 (−76.2, −56.8)
**Iraq**	6806.1 (5210.1, 8878.9)	14.7 (11.4, 19.0)	−40.6 (−46.4, −34.4)	9143.9 (7297.9, 11123.7)	23.4 (18.7, 28.3)	−42.7 (−46.1, −39.0)	15933.2 (10346.9, 21357.1)	38.9 (25.2, 52.0)	−52.4 (−64.6, −33.4)	244.5 (154.0, 325.1)	0.7 (0.4, 0.9)	−41.3 (−55.3, −22.0)
**Jordan**	2155.2 (1635.6, 2829.3)	17.3 (13.1, 22.7)	−37.1 (−43.3, −31.4)	3196.0 (2608.3, 3894.2)	26.7 (22.0, 32.4)	−40.2 (−43.4, −36.6)	4179.4 (2982.7, 5489.3)	36.7 (26.2, 47.8)	−54.2 (−64.2, −40.3)	64.4 (44.3, 82.9)	0.7 (0.5, 0.8)	−49.2 (−59.8, −33.8)
**Kuwait**	500.5 (377.6, 660.3)	15.1 (11.2, 20.4)	−40.5 (−46.1, −34.6)	1132.2 (925.5, 1373.6)	22.8 (18.7, 27.7)	−42.1 (−45.8, −38.3)	592.3 (494.9, 690.0)	16.8 (14.0, 19.5)	−78.4 (−82.0, −74.5)	10.3 (8.5, 12.3)	0.3 (0.3, 0.4)	−76.1 (−80.1, −71.7)
**Lebanon**	914.8 (705.3, 1192.3)	19.8 (15.1, 26.2)	−34.3 (−39.7, −28.0)	1749.4 (1423.3, 2120.7)	30.1 (24.4, 36.7)	−37.6 (−40.7, −34.2)	1093.2 (692.0, 1358.0)	21.4 (13.5, 27.2)	−53.2 (−63.6, −36.6)	24.5 (14.4, 29.7)	0.4 (0.3, 0.5)	−44.3 (−55.8, −26.9)
**Libya**	871.2 (686.9, 1101.9)	15.7 (12.1, 20.1)	−27.9 (−34.8, −20.8)	1763.9 (1450.4, 2111.5)	24.8 (20.6, 29.6)	−30.0 (−33.4, −26.3)	2893.3 (1649.7, 3876.0)	53.8 (31.2, 72.4)	−13.3 (−40.4, 25.1)	47.0 (25.6, 65.3)	0.9 (0.5, 1.2)	−6.2 (−33.8, 36.1)
**Morocco**	5776.7 (4401.3, 7315.8)	16.7 (12.7, 21.3)	−37.6 (−43.4, −31.2)	10147.9 (8259.6, 12334.1)	26.9 (22.0, 32.7)	−39.6 (−43.5, −35.6)	12721.7 (7476.6, 17376.7)	37.6 (21.9, 51.4)	−65.6 (−75.3, −45.2)	211.4 (120.0, 280.9)	0.6 (0.4, 0.8)	−55.4 (−66.6, −35.9)
**Oman**	535.4 (389.6, 742.0)	12.3 (9.0, 17.3)	−31.9 (−38.0, −25.8)	871.6 (710.6, 1065.5)	18.8 (15.4, 22.8)	−36.3 (−39.0, −33.5)	490.1 (301.0, 619.7)	11.9 (7.3, 14.9)	−53.2 (−66.7, −34.8)	7.7 (4.3, 9.9)	0.2 (0.1, 0.3)	−43.5 (−60.1, −20.8)
**Palestine**	1002.7 (739.9, 1327.9)	16.2 (12.1, 21.3)	−29.3 (−35.8, −23.3)	1196.7 (968.5, 1452.2)	25.8 (21.0, 31.0)	−32.0 (−35.3, −28.9)	1213.1 (769.4, 1567.6)	22.9 (14.4, 29.0)	−53.6 (−64.2, −38.4)	18.0 (10.8, 22.9)	0.4 (0.3, 0.5)	−45.1 (−56.0, −29.7)
**Qatar**	270.2 (204.6, 349.6)	13.4 (9.7, 18.0)	−46.1 (−51.7, −39.9)	624.7 (505.4, 760.2)	20.0 (16.3, 24.2)	−52.2 (−55.2, −49.5)	247.3 (160.3, 331.8)	10.9 (7.3, 14.4)	−52.1 (−67.6, −32.3)	4.0 (2.3, 5.5)	0.3 (0.2, 0.3)	−42.5 (−62.9, −13.8)
**Saudi Arabia**	4555.2 (3579.0, 5745.5)	15.4 (11.8, 19.8)	−21.9 (−29.0, −12.3)	9487.0 (7563.8, 11499.9)	24.1 (19.3, 29.2)	−26.9 (−30.5, −23.4)	15681.2 (9990.6, 21686.9)	41.3 (26.6, 55.6)	−58.3 (−70.9, −38.4)	299.2 (186.5, 416.7)	1.0 (0.6, 1.3)	−41.6 (−58.1, −18.9)
**Sudan**	8522.3 (6633.0, 11198.7)	15.5 (12.2, 20.1)	−42.9 (−49.6, −35.3)	10014.1 (8140.5, 12136.4)	25.5 (21.0, 30.6)	−43.1 (−48.2, −37.4)	33439.8 (16069.9, 46257.2)	64.5 (31.4, 88.7)	−67.4 (−78.9, −36.2)	423.4 (205.3, 586.0)	0.9 (0.5, 1.3)	−63.2 (−75.9, −33.2)
**Syrian Arab Republic**	2230.6 (1726.0, 2893.5)	17.1 (13.4, 21.9)	−42.1 (−47.4, −36.8)	3843.3 (3114.5, 4640.1)	27.0 (21.9, 32.5)	−44.3 (−48.1, −40.6)	5693.0 (3846.8, 8038.8)	44.6 (30.2, 62.8)	−53.8 (−65.9, −32.5)	112.7 (70.0, 151.1)	1.0 (0.6, 1.3)	−40.0 (−56.2, −14.2)
**Tunisia**	1577.2 (1215.8, 2040.9)	15.9 (12.2, 20.7)	−37.5 (−42.4, −32.1)	3080.0 (2534.9, 3686.1)	24.8 (20.4, 29.7)	−40.0 (−43.5, −36.6)	3046.7 (1829.3, 4162.7)	29.5 (17.3, 40.6)	−64.6 (−76.0, −47.9)	57.7 (33.4, 79.6)	0.5 (0.3, 0.7)	−53.3 (−67.5, −34.7)
**Turkey**	9218.5 (7061.5, 11972.7)	13.2 (10.0, 17.3)	−36.7 (−43.2, −30.7)	18525.6 (15181.4, 22287.2)	20.8 (17.1, 25.0)	−40.5 (−43.6, −37.3)	15925.5 (10122.9, 19954.8)	23.0 (14.8, 29.5)	−51.5 (−65.7, −33.5)	318.0 (195.2, 390.2)	0.4 (0.3, 0.5)	−41.9 (−56.4, −24.3)
**United Arab Emirates**	830.7 (641.1, 1093.3)	16.0 (12.1, 21.5)	−28.2 (−33.6, −22.0)	2637.1 (2157.9, 3190.5)	25.0 (20.6, 30.3)	−31.7 (−35.4, −28.5)	1013.3 (641.8, 1267.5)	16.2 (10.6, 20.0)	−60.4 (−71.4, −40.7)	17.2 (9.5, 21.8)	0.4 (0.2, 0.5)	−49.1 (−63.4, −25.2)
**Yemen**	7147.3 (5475.6, 9364.9)	16.1 (12.5, 20.8)	−32.9 (−39.2, −26.1)	7970.0 (6458.7, 9659.8)	26.8 (22.0, 32.1)	−33.2 (−37.8, −29.0)	22955.9 (12214.1, 30721.4)	55.3 (29.4, 73.2)	−57.1 (−70.1, −23.1)	291.6 (151.8, 388.6)	0.8 (0.4, 1.1)	−50.8 (−65.3, −21.8)

Abbreviations: DAxLY, disability‐adjusted life year; Pcs, percent changes; UI, uncertainty interval.

### 3.2 Epidemiology at the Country Levels

**Figure 1:**
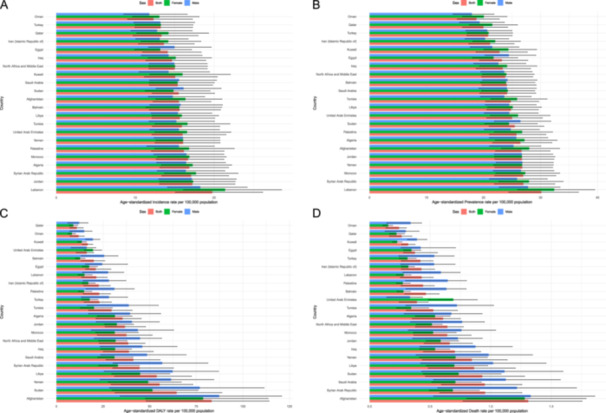
Age‐standardized incidence (A), point prevalence (B), DALY (C), and death rates (D) for pulmonary aspiration and foreign body in the airway (per 100,000 population) in the Middle East and North Africa region in 2021, by sex and country. DALY = disability‐adjusted‐life‐year. (Generated from data available from http://ghdx.healthdata.org/gbd-results-tool).

The highest age‐standardized incidence rates of PAFBA among MENA countries in 2021 were recorded in Lebanon, with 19.8 per 100,000 (15.1 to 26.2), and Jordan, at 17.3 per 100,000 (13.1 to 22.7). Conversely, the lowest incidence rates were observed in Oman (12.3; 9.0 to 17.3) and Turkey (13.2; 10.0 to 17.3). Notably, from 1990 to 2021, all countries saw a reduction in age‐standardized incidence rates, with the largest decreases in Iran, showing a 50.2% reduction (−53.7 to −46.9), and Qatar, with a 46.1% decrease (−51.7 to −39.9) (Table 1, Figure 1A, and Figure S1).

For prevalence, Lebanon had the highest age‐standardized point prevalence of PAFBA in 2021, with 30.1 per 100,000 (24.4 to 36.7), followed by Syria with 27.0 per 100,000 (21.9 to 32.5). The lowest prevalence rates were seen in Oman at 18.8 per 100,000 (15.4 to 22.8), and Qatar at 20.0 per 100,000 (16.3 to 24.2). From 1990 to 2021, age‐standardized point prevalence decreased across all nations, with Iran and Qatar experiencing the most substantial reductions, by 52.2% (−56.1 to −48.5) and 52.2% (−55.2 to −49.5), respectively (Table 1, Figure 1B, and Figure S2).

In terms of DALYs, Afghanistan had the highest age‐standardized DALY rate due to PAFBA in 2021, with 83.2 per 100,000 (48.1 to 114.1), followed by Sudan with 64.5 per 100,000 (31.4 to 88.7). The lowest DALY rates due to PAFBA were seen in Qatar at 10.9 per 100,000 (7.3 to 14.4) and Oman at 11.9 per 100,000 (7.3 to 14.9). Over the period from 1990 to 2021, DALY rates decreased across the MENA region and countries, with the largest declines seen in Iran at 79.4% (−84.3 to −68.1) and Egypt at 78.8% (−84.3 to −60.4) (Table 1, Figure 1C, and Figure S3).

Regarding mortality, the highest age‐standardized death rates due to PAFBA in 2021 were found in Afghanistan, with 1.3 per 100,000 (0.8 to 1.8), and Syria, with 1.0 per 100,000 (0.6 to 1.3). The nations with the lowest death rates were Oman (0.2; 0.1 to 0.3) and Qatar (0.3; 0.2 to 0.3). From 1990 to 2021, mortality rates among both sexes combined decreased across all MENA nations, with the most pronounced reductions seen in Egypt, showing a 79.0% decrease (−84.3 to −50.9), and Kuwait with a 76.1% decrease (−80.1 to −71.7) (Table 1, Figure 1D, and Figure S4).

### 3.3 Age and Sex Patterns

**Figure 2:**
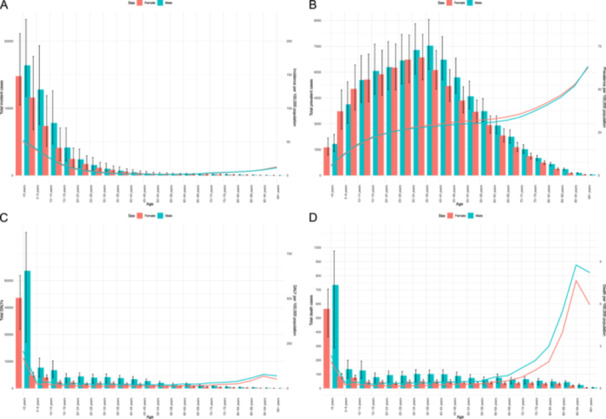
Number of incident cases and incidence rate (A), number of prevalent cases and prevalence (B), number of DALYs and DALY rate (C), and the number of deaths and death rate (D) for pulmonary aspiration and foreign body in the airway (per 100,000 population) in the Middle East and North Africa region, by age and sex in 2021. DALY = disability‐adjusted‐life‐year. (Generated from data available from http://ghdx.healthdata.org/gbd-results-tool).

The highest incident cases and incidence rates for PAFBA were observed in the < 5‐year age group in the MENA region in 2021. Moreover, males had higher incident cases than females in 2021 (Figure 2A). Regarding prevalence, the prevalent cases increased up to the 35–39 age group in both males and females, followed by a decrease after that. The point prevalence increased with age up to the 95+ age group. Prevalent cases were higher in males than females in all age groups (Figure 2B). The DALY counts were highest in the < 5‐year age group. The DALY rates had two peaks in the < 5 and 90–94 age groups. The DALY counts and rates were higher in males (Figure 2C). Similarly, death counts were highest in children under‐5 years old. The death rates also showed two peaks in children under‐5 years and adults aged 90–94 years. The number of deaths and death rates were higher in males (Figure 2D).

### 3.4 Association With Socio‐Demographic Index

**Figure 3:**
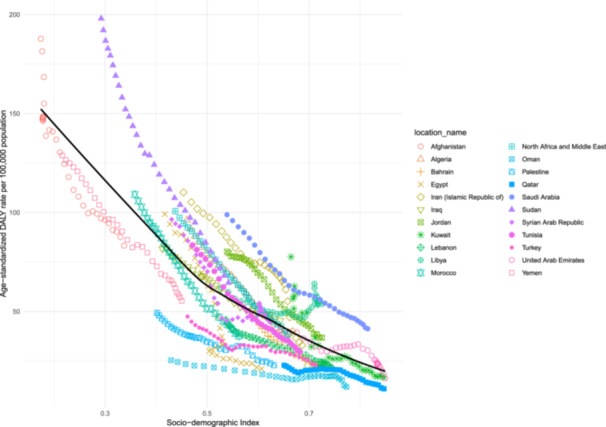
Age‐standardized DALY rates of pulmonary aspiration and foreign body in the airway for the 21 countries between 1990 and 2021, by SDI; Expected values based on the SDI and the condition rates in all locations are shown as the black line. Each point shows the observed age‐standardized DALY rate for each country from 1990 to 2021. DALY = disability‐adjusted‐life‐year. SDI = socio‐demographic index (Generated from data available from http://ghdx.healthdata.org/gbd-results-tool).

There was a negative association between age‐standardized DALY rates of PAFBA in the MENA region and SDI, as countries with improved conditions had a lower burden. Countries, such as Saudi Arabia, Sudan, and Jordan had an age‐standardized DALY rate higher than expected for PAFBA in MENA. However, Qatar, Oman, Palestine, Yemen, and Turkey had lower than expected age‐standardized DALY rates (Figure 3).

## 4 Discussion

Our findings showed a decreasing trend of PAFBA in the MENA region and countries between 1990 and 2021, with Iran showing the greatest decrease in incidence, prevalence, and DALY rates. By country, Lebanon had the largest rates of incidence and prevalence, while Afghanistan had the highest DALY and death rates. Under‐5 children and geriatric populations had a higher reported burden of PAFBA in MENA. Furthermore, there were higher DALY rates in countries with lower levels of socioeconomic development.

Our study revealed 38.9%, 40.9%, 61.8%, and 55.6% decreases in the incidence, prevalence, DALY, and death rates of PAFBA over 1990–2021 in the MENA region. Similarly, the average annual percent changes for incidence and mortality of foreign body aspiration in the MENA region were −0.05 (95% confidence interval [95% CI]: −0.06, −0.05) and −1.83 (95% CI: −1.90, −1.75) from 1990 to 2019, respectively [5]. In 2019, the age‐standardized incidence and death rates of foreign body aspiration were 767.7 and 0.9 per 100,000 in MENA, respectively, both lower than the global rates in the same year [5]. The decrease in the burden of PAFBA can be explained by several reasons. First, there has been improvements in healthcare access and quality globally and in the MENA region. Accordingly, the article by Hanifiha et al. showed that the quality of care index, a secondary metric developed to assess quality access, showed 9.4% improvements for foreign body among children and adolescents from 1990 to 2017 globally [10]. Second, there have been improvements in imaging techniques, access, and teleservices in the MENA region, which can be effective for early detection and management of PAFBA, and thus might lead to a decrease in its burden [11]. Third, there might be increases in education and awareness of parents regarding the prevention and clinical manifestations of PAFBA, which can lead to early diagnosis and reduce complications. In this regard, informing parents about age‐appropriate foods, promoting safe eating practices, ensuring a secure environment, and offering guidance on routine child care, along with equipping parents with essential first aid skills should be considered [12]. Preliminary data from GBD 2023 indicate continued alignment with these trends, with no significant shifts in PAFBA burden that would alter our conclusions [13].

At the country level, Iran had the greatest decreases in the age‐standardized rates of incidence, prevalence, and DALYs, while Egypt had the greatest decrease in death rates. Although Lebanon had the highest age‐standardized rates of incidence and point prevalence, the highest death and DALY rates were observed in Afghanistan. The significant decreases in incidence, prevalence, and DALY rates in Iran, and mortality rates in Egypt, may be due to improved healthcare infrastructure, emergency response systems, and better access to care with low costs and high quality [14]. In addition, these countries may have benefited from public health campaigns focused on reducing the risk of foreign body aspiration, especially among young children [15]. Lebanon's elevated incidence and prevalence rates may be attributed to cultural factors, such as the use of metallic pins for headscarves among women, which increases aspiration risks, as well as potentially higher reporting due to better diagnostic capabilities in a middle‐SDI setting [6]. In contrast, Afghanistan and Sudan may have higher exposure to risk factors associated with PAFBA due to lifestyle and environmental conditions, such as the widespread use of small and aspiratable objects in daily life. Additionally, Afghanistan's high DALY and death rates are likely influenced by its low SDI, ongoing conflicts that disrupt healthcare delivery, limited access to bronchoscopy and emergency care, and broader challenges in child health outcomes [10]. Moreover, Afghanistan had the lowest quality of care index for foreign body aspiration among children and adolescents in the MENA region in 2017, representing limited healthcare infrastructure and ongoing economic challenges [10].

The results showed that the greatest incidence, DALYs, and mortalities were among males, and those aged < 5 years and 90–94 years. The results are generally in accordance with the results of the prior study based on GBD 2019 [5]. In addition, the global DALY rates of foreign body aspiration were higher among males than females in 2019 among children aged < 5 years (335.5 vs. 299.2 per 100,000) [7]. The study by Parvar and colleagues involving more than 14 thousand participants also showed that above 75% of foreign body aspirations occurred in children under‐2‐years old [16]. For children under 5 years, the frequent occurrence of PAFBA can be explained by developmental factors, such as an increased tendency to explore objects by putting them in their mouths, as well as incomplete motor skills and airway protection mechanisms. In older adults, particularly those aged 90–94, the incidence may be linked to factors such as frailty, reduced swallowing reflex, and a higher prevalence of chronic health conditions, which increase the risk of aspiration and related complications. These findings underscore the critical need for targeted prevention strategies focused on these high‐risk age and sex groups, especially in pediatric and elderly populations, to reduce the burden of PAFBA in this region.

We observed that with increases in socioeconomic development, measured by SDI, the age‐standardized DALY rates decreased. In 2019, the middle SDI quintile had the greatest DALY rate in children < 5 years globally [7]. The differences between the mentioned study and ours can be due to the inclusion of different populations (under‐5 children vs. all age groups), different locations (global vs. MENA), and different times (2019 vs. 2021). One of the reasons for a higher burden in low SDI countries can be limited access to medical imaging [17]. This can lead to delayed diagnosis, often resulting in more severe outcomes, increased complications, and higher mortality and DALY rates. In addition to limited access to medical imaging, a higher PAFBA burden in low SDI countries is influenced by factors such as inadequate healthcare infrastructure, a shortage of skilled professionals, poor health education, and economic constraints. To improve access to medical imaging in less developed countries, a strategy has been proposed, focusing on a model for organizing imaging services through regional Centers of Excellence, to help access to diagnostic technologies. Additionally, the plan emphasizes the importance of using information technology and artificial intelligence in imaging to improve diagnostic accuracy and reduce the burden of diseases like PAFBA [18].

The strength of this study is the use of comprehensive data on the epidemiology of PAFBA, by age and sex. Moreover, we specifically focused PAFBA, not overall foreign body aspiration and its types, in this region to provide information for health policymaking and resource allocation. However, we should acknowledge several limitations. First, there is a possibility of underreporting of PAFBA cases because of low educational levels and limited access to appropriate diagnostic modalities, especially in low‐ and middle‐income countries in this region. Furthermore, many individuals with mild or asymptomatic PAFBA may avoid medical care, making early detection challenging. As a result, modelling techniques were used to estimate the epidemiological metrics of PAFBA at the regional and national levels. To address this, 95% UIs were used to represent the uncertainty in the results. This underreporting and data sparsity in MENA contribute to wider and sometimes overlapping UIs, potentially limiting the ability to detect subtle differences. Future studies could mitigate this through enhanced surveillance systems, such as improved vital registration and integration of electronic health records, to narrow UIs and strengthen statistical inferences. Second, the reliability of GBD findings is associated with the quality and availability of the data used in its models. Without more detailed data on social, economic, and individual factors specific to the MENA region, results may have some bias. Therefore, addressing these limitations will require broader data collection to support a more comprehensive and accurate assessment of PAFBA burden in this region. Regarding generalizability, our findings on PAFBA burden in MENA may not fully extend to the global population due to region‐specific factors, such as cultural practices and varying healthcare access influenced by conflicts and economic disparities. However, main patterns, including declines in age‐standardized incidence, prevalence, DALY, and death rates; peaks in burden among under‐5 children and the elderly; male predominance; and a negative correlation with SDI, are aligned with global GBD analyses [5, 7]. These similarities suggest applicability to other low‐ and middle‐SDI regions, but caution is advised for high‐SDI areas with different risk profiles and better preventive measures. Broader global comparisons would benefit from integrated multi‐regional studies.

## 5 Conclusions

Over the past 32 years, the MENA region has seen a decline in the burden of PAFBA; however, mortality rates and DALYs remain high across its 21 countries. The impact is particularly severe in countries like Lebanon and Afghanistan, among children, and in less developed areas. To further reduce the burden of PAFBA, implementing educational programs to raise public awareness, especially among parents, alongside advancements in diagnostic and therapeutic interventions should be prioritized in the region.

## Author Contributions

**Fereshteh Kimia:** writing – original draft, writing – review and editing, methodology, conceptualization, software, data curation, investigation, validation, formal analysis, visualization. **Fatemeh Roodneshin:** writing – original draft, writing – review and editing, methodology, project administration, software. **Mahtab Poorzamany Nejat Kermany:** writing – original draft, writing – review and editing, methodology, project administration, software. **Hamidreza Samadpour:** writing – original draft, writing – review and editing, formal analysis, visualization, software, data curation. **Nima Saeedi:** writing – original draft, writing – review and editing, methodology, project administration, software. **Mina Vishte:** writing – original draft, writing – review and editing, methodology, conceptualization, software, data curation, investigation, validation, formal analysis, visualization.

## Funding

The authors have nothing to report.

## Ethics Statement

The study did not require formal ethics approval as determined by the Ethics Committee of Shahid Beheshti University of Medical Sciences. The committee waived the need for approval because the research involved only secondary data analysis of de‐identified human data. The data used in the study were entirely anonymized prior to access, ensuring that no identifying information about individuals was available to the researchers. Since the study did not involve any interventions, direct interactions with participants, or the collection of new data from individuals, it was deemed exempt from formal ethics review under national research regulations. No vulnerable populations were involved, and there were no invasive procedures or risk to participants' privacy or well‐being. Additionally, there were no fatalities or significant health outcomes reported for the individuals whose data were analyzed. This approach complies fully with national and institutional ethical standards governing the use of anonymized secondary data for research purposes. As the data were already anonymized and publicly accessible, no reference number was issued for this exemption.

## Conflicts of Interest

The authors declare no conflicts of interest.

## Transparency Statement

The lead author Mina Vishte affirms that this manuscript is an honest, accurate, and transparent account of the study being reported; that no important aspects of the study have been omitted; and that any discrepancies from the study as planned (and, if relevant, registered) have been explained.

## Supporting information


**Figure S1:** The percentage change in the age‐standardized incidence rate of pulmonary aspiration and foreign body in the airway in the Middle East and North Africa region from 1990 to 2021, by sex and country.
**Figure S2:** The percentage change in the age‐standardized point prevalence of pulmonary aspiration and foreign body in the airway in the Middle East and North Africa region from 1990 to 2021, by sex and country.
**Figure S3:** The percentage change in the age‐standardized DALY rates of pulmonary aspiration and foreign body in the airway in the Middle East and North Africa region from 1990 to 2021, by sex and country.
**Figure S4:** The percentage change in the age‐standardized death rates of pulmonary aspiration and foreign body in the airway in the Middle East and North Africa region from 1990 to 2021, by sex and country.

## Data Availability

The data used for these analyses are all publicly available at https://vizhub.healthdata.org/gbd-results/.
