# Supplementary material for: The Epidemiology and Socioeconomic Associates of Pulmonary Aspiration and Foreign Body in the Airway in the Middle East and North Africa Region From 1990 to 2021: A Descriptive Epidemiological Study
Source: Health Sci Rep. 2026 May 9;9(5):e72311. doi: 10.1002/hsr2.72311 (PMC13156812; doi:10.1002/hsr2.72311)
Supplement: Supplementary file 1 — Figure S1: The percentage change in the age‐standardized incidence rate of pulmonary aspiration and foreign body in the airway in the Middle East and North Africa region from 1990 to 2021, by sex and country. Figure S2: The percentage change in the age‐standardized point prevalence of pulmonary aspiration and foreign body in the airway in the Middle East and North Africa region from 1990 to 2021, by sex and country. Figure S3: The percentage change in the age‐standardized DALY rates of pulmonary aspiration and foreign body in the airway in the Middle East and North Africa region from 1990 to 2021, by sex and country. Figure S4: The percentage change in the age‐standardized death rates of pulmonary aspiration and foreign body in the airway in the Middle East and North Africa region from 1990 to 2021, by sex and country. [file HSR2-9-e72311-s001.pdf]

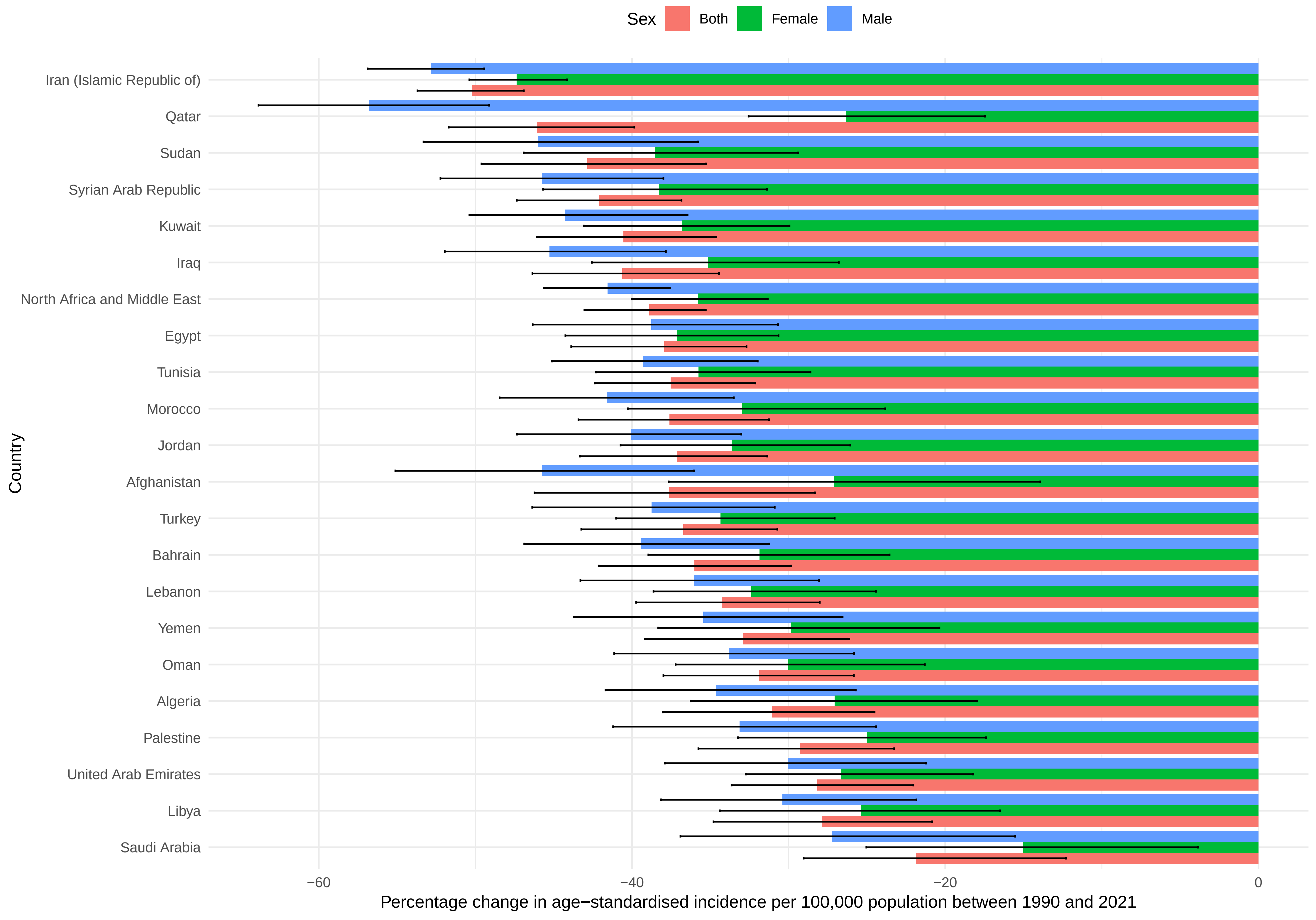

Sex
Both
Female
Male
Iran (Islamic Republic of)
Qatar
Sudan
Syrian Arab Republic
Kuwait
Iraq
North Africa and Middle East
Egypt
Tunisia
Morocco
Jordan
Afghanistan
Turkey
Bahrain
Lebanon
Yemen
Oman
Algeria
Palestine
United Arab Emirates
Libya
Saudi Arabia
Country
−60
−40
−20
0
Percentage change in age−standardised incidence per 100,000 population between 1990 and 2021

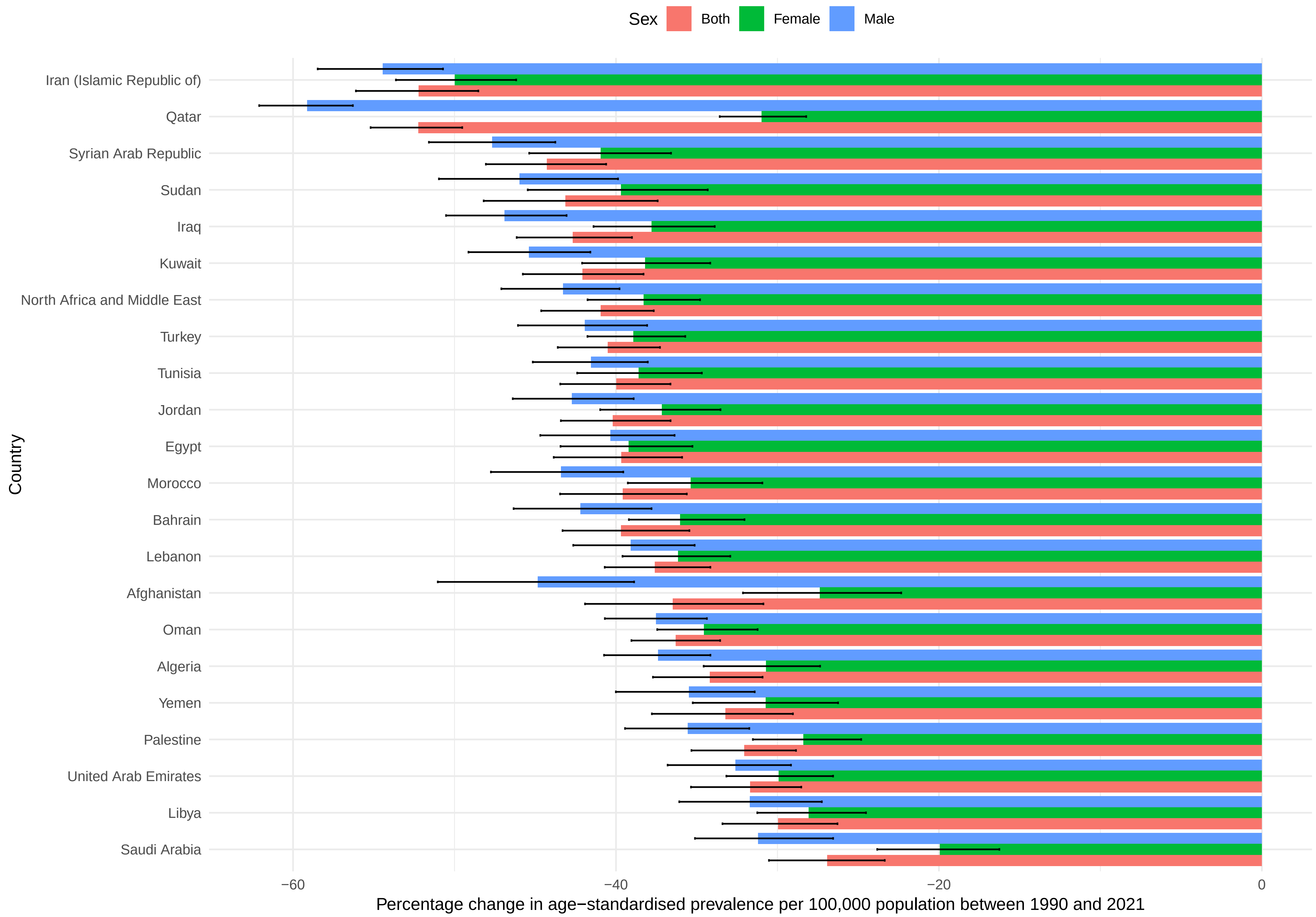

Sex
Both
Female
Male
Iran (Islamic Republic of)
Qatar
Syrian Arab Republic
Sudan
Iraq
Kuwait
North Africa and Middle East
Turkey
Tunisia
Jordan
Egypt
Morocco
Bahrain
Lebanon
Afghanistan
Oman
Algeria
Yemen
Palestine
United Arab Emirates
Libya
Saudi Arabia
Country
−60
−40
−20
0
Percentage change in age−standardised prevalence per 100,000 population between 1990 and 2021

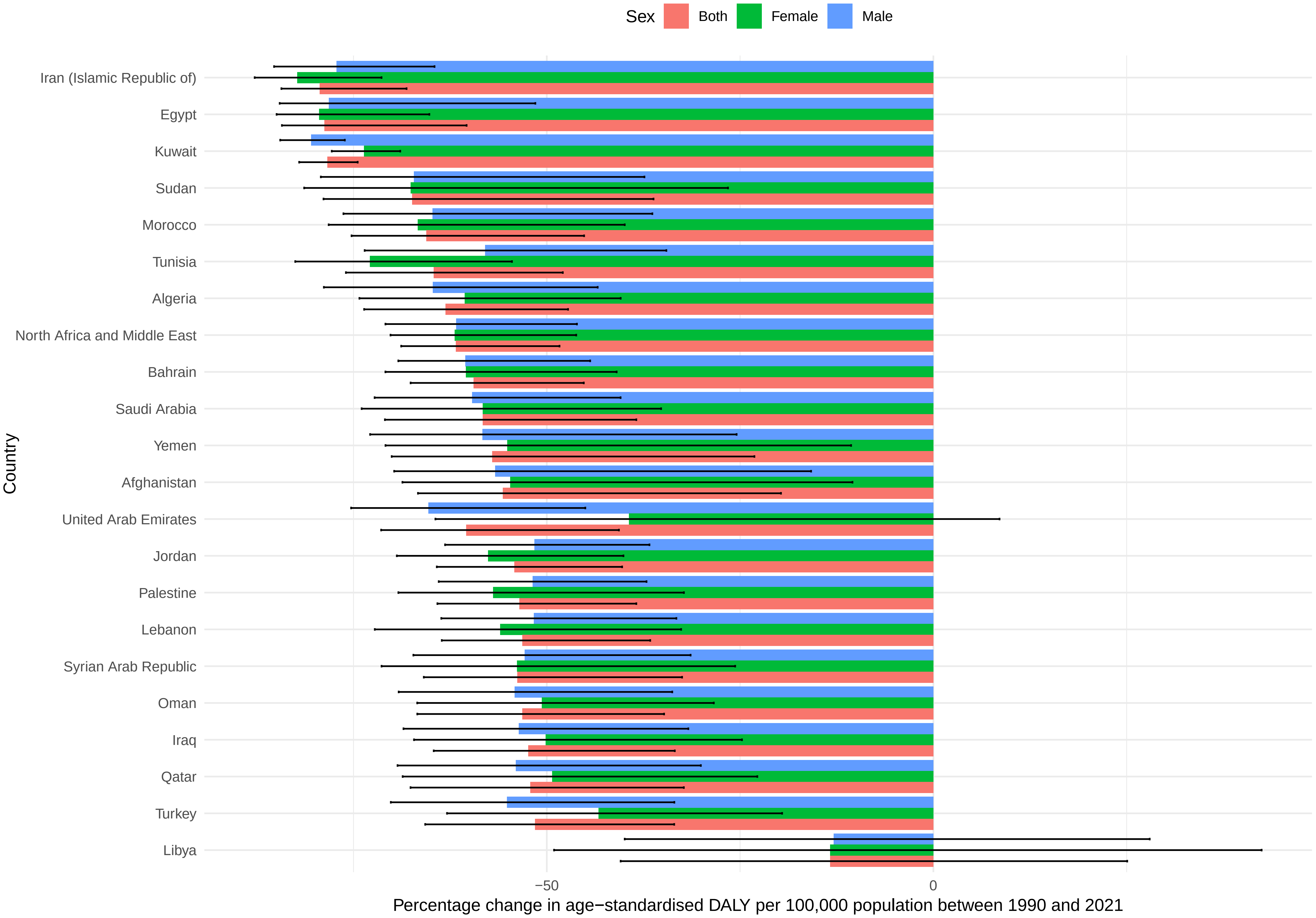

Sex
Both
Female
Male
Iran (Islamic Republic of)
Egypt
Kuwait
Sudan
Morocco
Tunisia
Algeria
North Africa and Middle East
Bahrain
Saudi Arabia
Yemen
Afghanistan
United Arab Emirates
Jordan
Palestine
Lebanon
Syrian Arab Republic
Oman
Iraq
Qatar
Turkey
Libya
Country
−50
0
Percentage change in age−standardised DALY per 100,000 population between 1990 and 2021

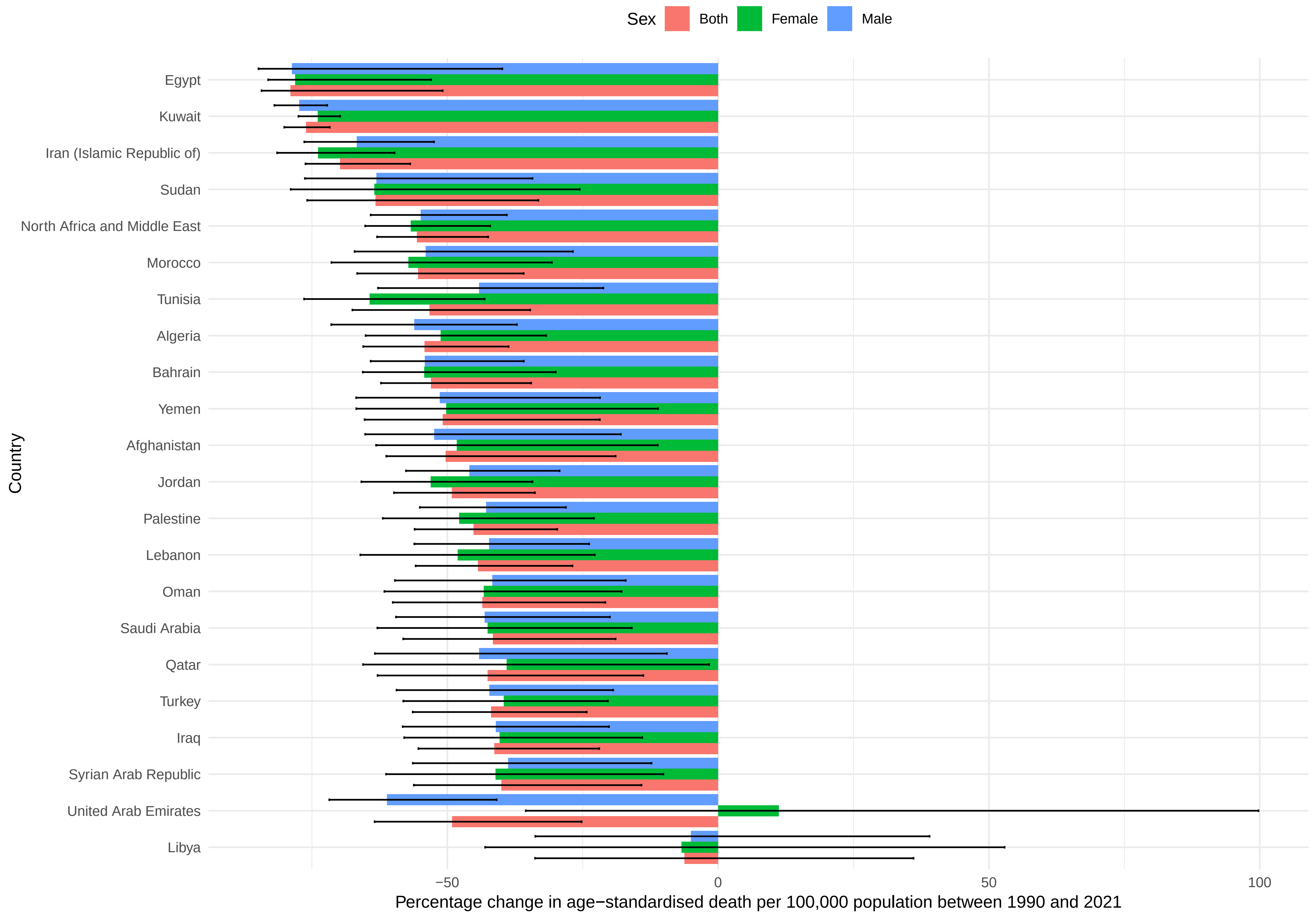

Sex
Both
Female
Male
Egypt
Kuwait
Iran (Islamic Republic of)
Sudan
North Africa and Middle East
Morocco
Tunisia
Algeria
Bahrain
Yemen
Afghanistan
Jordan
Palestine
Lebanon
Oman
Saudi Arabia
Qatar
Turkey
Iraq
Syrian Arab Republic
United Arab Emirates
Libya
Country
−50
0
50
100
Percentage change in age−standardised death per 100,000 population between 1990 and 2021
